# Editorial: Role of plant cell wall in biotic and abiotic stress resistance

**DOI:** 10.3389/fpls.2025.1617371

**Published:** 2025-05-19

**Authors:** Seungmin Son, Jong Hee Im, Jae-Heung Ko, Kyung-Hwan Han

**Affiliations:** ^1^ National Institute of Agricultural Sciences, Rural Development Administration, Jeonju, Republic of Korea; ^2^ Interdisciplinary Graduate Program in Advanced Convergence Technology and Science, Jeju National University, Jeju, Republic of Korea; ^3^ Department of Convergent Biotechnology and Advanced Materials Science, College of Life Science and Graduate School of Green-Bio Scinece, Kyung Hee University, Yongin-si, Gyeonggi-do, Republic of Korea; ^4^ Department of Horticulture, Michigan State University, East Lansing, MI, United States; ^5^ Department of Forestry, Michigan State University, East Lansing, MI, United States

**Keywords:** cell wall, environmental stimuli, MYC2, oligogalacturonide, pectin methylesterase inhibitor, wall-associated kinase

The plant cell wall functions as a dynamic and multifaceted interface between the plant and its external environment, critically contributing to the maintenance of structural integrity, regulation of growth and development, and facilitation of adaptive responses to both biotic and abiotic stresses. Extensive research has progressively unraveled the intricate molecular mechanisms underlying cell wall remodeling, immune system activation, and resilience to environmental challenges, highlighting the complexity of the regulatory networks that orchestrate these processes. These sophisticated signaling pathways and modulatory mechanisms are fundamental to plant survival, enabling them to withstand and adapt to continuously fluctuating external conditions. The Research Topic, *Role of Plant Cell Wall in Biotic and Abiotic Stress Resistance*, comprises seven scholarly contributions that collectively enhance our comprehension of these intricate processes, encompassing a broad spectrum of aspects, including comprehensive gene family characterization, molecular and physiological adaptations to abiotic stress, and the intricate signaling cascades involved in plant immunity.

The two review articles featured in this Research Topic provide in-depth overviews of important gene families and molecular components that regulate cell wall signaling and integrity, underscoring the plant cell wall’s role as a dynamic regulator of stress responses beyond its structural function. Harvey et al. focuses on the wall-associated kinase (WAK) and WAK-like (WAKL) families across diverse plant species, providing comprehensive information on their function as transmembrane pectin receptors involved in cell wall integrity and stress responses. Their analysis highlights inconsistencies in current classification methodologies and emphasizes the need for a standardized framework for characterizing *WAK/WAKL* genes, which is essential for elucidating their precise roles in plant development and adaptation to environmental challenges. The second review highlights oligogalacturonides (OGs), a class of cell wall-derived damage-associated molecular patterns, which are released upon pathogen invasion or mechanical injury and serve as crucial immune modulators by activating intracellular defense pathways (Esposti et al.). While OGs contribute to enhanced plant resistance, their excessive accumulation can lead to immune hyperactivation, necessitating finely tuned regulatory mechanisms to balance defense responses. Notably, the potential application of OGs in sustainable agriculture is particularly promising, as they offer an environmentally friendly alternative to synthetic pesticides that can improve crop resilience without ecological drawbacks.

The research article on the pectin methylesterase inhibitor (PMEI) family in watermelon (*Citrullus lanatus*) presents the identification and characterization of 60 *ClPMEI* genes, categorizing them into three subfamilies (Zhang et al.). Analysis of *cis*-regulatory elements and gene expression profiles indicates that *ClPMEI* genes are responsive to low-temperature and drought stress conditions. These findings suggest that *ClPMEI* genes play a functional role in the molecular pathways underlying watermelon adaptation to abiotic stress, offering new targets for improving stress resilience in watermelon through genetic modifications. In the context of secondary metabolites, the research on UV-induced reactive oxygen species and 3-deoxyanthocyanidin biosynthesis in black sorghum pericarp (*Sorghum bicolor*) provides valuable insights into the interplay between light exposure, oxidative stress, and flavonoid pathway activation, providing a foundational framework for future studies aimed at identifying key regulators and understanding the regulatory mechanisms governing the black sorghum pericarp trait (Schumaker et al.). Moreover, the study on sugarcane leaves exposed to elevated CO₂ (eCO₂) and drought stress investigates the impact of these factors on biomass accumulation and cell wall composition (Araujo et al.). Findings reveal that eCO₂ enhances leaf biomass production and mitigates drought-induced reductions, highlighting its potential to alleviate the adverse effects of water deficit stress. Notably, alterations in arabinosylation, fucosylation, and mixed-linkage glucan composition suggest a strategic remodeling of cell wall architecture to enhance resilience under stress conditions.

While this Research Topic primarily focuses on plant cell walls, it also encompasses a study on the phytopathogenic fungus, unveiling the crucial function of nucleolin MoNsr1 in regulating fungal cell wall biogenesis and stress resilience in the rice blast pathogen *Magnaporthe oryzae* (Zhang et al.). Given the indispensable role of cell walls in host-pathogen interactions, these findings offer a complementary perspective on cell wall functions in plant-associated fungi. By elucidating the contribution of MoNsr1 to fungal virulence, this study not only advances our comprehension of pathogenic mechanisms but also suggests potential molecular targets for developing strategies to control *M. oryzae*, which is a devastating rice pathogen responsible for significant global yield losses.

Finally, the opinion article on MYELOCYTOMATOSIS 2 (MYC2) signaling in cell wall modulation provides insight into the intricate regulatory network governing this process (Im and Son). As a central regulator of the jasmonic acid signaling pathway, MYC2 orchestrates interactions with multiple transcription factors and hormone signaling cascades to fine-tune cell wall remodeling in response to environmental cues. The proposed development of synthetic promoters and engineered MYC2 variants presents innovative strategies to enhance secondary cell wall formation, offering potential avenues for improving plant stress resilience and optimizing biomass production.

Collectively, this Research Topic highlights the pivotal roles of the cell wall and the complex, multifaceted molecular mechanisms governing its remodeling, emphasizing its significance in maintaining cellular integrity and facilitating plant adaptive responses to environmental perturbations. Furthermore, by elucidating the regulatory networks that mediate these intricate processes, the featured studies enhance our understanding of cell wall dynamics at both molecular and physiological levels. Additionally, they provide valuable insights into potential strategies for improving crop resilience through targeted genetic modifications and biotechnological interventions, thereby contributing to the advancement of sustainable agricultural practices in the face of escalating environmental challenges.

